# Defining NAD(P)(H) Catabolism

**DOI:** 10.3390/nu15133064

**Published:** 2023-07-07

**Authors:** Jyothi Dhuguru, Ryan W. Dellinger, Marie E. Migaud

**Affiliations:** 1 Department of Pharmacology, Mitchell Cancer Institute, College of Medicine, University of South Alabama, 1660 Springhill Avenue, Mobile, AL 36604, USA; jyothidhuguru@southalabama.edu; 2 Elysium Health, New York, NY 10013, USA; ryan@elysiumhealth.com

**Keywords:** NAD^+^ metabolism, NAD(P)(H) catabolism, nicotinamide, niacin, methyl-nicotinamide, pyridone

## Abstract

Dietary vitamin B3 components, such as nicotinamide and nicotinic acid, are precursors to the ubiquitous redox cofactor nicotinamide adenine dinucleotide (NAD^+^). NAD^+^ levels are thought to decline with age and disease. While the drivers of this decline remain under intense investigation, strategies have emerged seeking to functionally maintain NAD^+^ levels through supplementation with NAD^+^ biosynthetic intermediates. These include marketed products, such as nicotinamide riboside (NR) and its phosphorylated form (NMN). More recent developments have shown that NRH (the reduced form of NR) and its phosphorylated form NMNH also increases NAD^+^ levels upon administration, although they initially generate NADH (the reduced form of NAD^+^). Other means to increase the combined levels of NAD^+^ and NADH, NAD(H), include the inhibition of NAD^+^-consuming enzymes or activation of biosynthetic pathways. Multiple studies have shown that supplementation with an NAD(H) precursor changes the profile of NAD(H) catabolism. Yet, the pharmacological significance of NAD(H) catabolites is rarely considered although the distribution and abundance of these catabolites differ depending on the NAD(H) precursor used, the species in which the study is conducted, and the tissues used for the quantification. Significantly, some of these metabolites have emerged as biomarkers in physiological disorders and might not be innocuous. Herein, we review the known and emerging catabolites of the NAD(H) metabolome and highlight their biochemical and physiological function as well as key chemical and biochemical reactions leading to their formation. Furthermore, we emphasize the need for analytical methods that inform on the full NAD(H) metabolome since the relative abundance of NAD(H) catabolites informs how NAD(H) precursors are used, recycled, and eliminated.

## 1. Introduction

**Figure 1:**
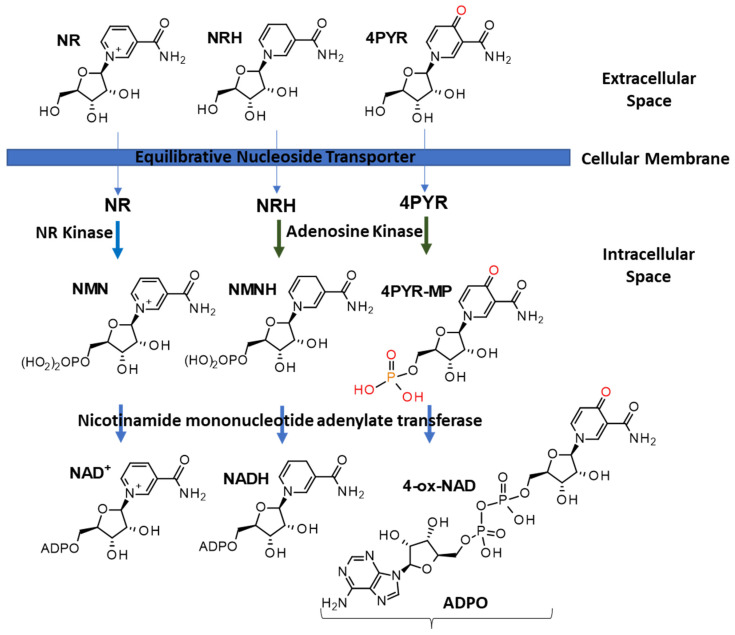
Schematic representation of the formation of intracellular nucleotides and dinucleotides derived from extracellular ribosylated precursors.

Dietary vitamin B3 is the naturally occurring source of nicotinamide adenine dinucleotide (NAD^+^) (Figure 1). NAD+ was discovered by Harden and Young [1,2] in 1906 and initially characterized by von Euler-Chelpin in 1929. Since then, NAD+ has been found to be a critical intracellular coenzyme [3] involved in the regulation of energy metabolism [4], as well as being vital to cellular events such as DNA repair, gene expression, oxidative stress, aging, and cell death [5]. NAD^+^ is an obligatory catalyst in energy production pathways such as glycolysis, TCA cycle, fatty acid oxidation, and oxidative phosphorylation, where it is the redox partner of NADH. NAD^+^ is the only precursor of NADP(H), the phosphorylated form of NAD(H). Furthermore, unlike NADH, NAD^+^ is also a substrate and co-substrate for NAD^+^-consuming enzymes. These enzymes include ADP-ribosyl cyclase, SARM-1, NAD hydrolases, mono(ADP-ribosyl)transferases, poly(ADP-ribose) polymerases, and sirtuins. These enzymes consume NAD^+^ with the release of nicotinamide. The activity of these enzymes is under the direct control of NAD^+^ levels and is unaffected by NADH [6]. In cells and tissues, perturbations in the NAD^+^ biosynthetic pathways, and over-activation of NAD^+^-consuming enzymes lead to NAD^+^ depletion [7,8]. Since NAD^+^ and its reduced form, NADH, are partners in intracellular redox reactions, perturbations in the NAD^+^ levels are predicted to affect NADH levels [9,10]. Similarly, reductive stress, whereby the NADH to NAD^+^ ratio is shifted towards NADH can affect the availability of NAD^+^ and thus that of NAD^+^-consuming enzymes [11,12,13]. 

## 2. NAD(P)(H) Metabolism

NAD(P)(H) biosynthesis: It has been observed by many that a decrease in NAD^+^ levels can negatively affect mitochondrial and cellular function [14,15]. Mounting evidence also supports that NAD^+^ levels decrease with aging and the timeline of such decrease appears to be gender-dependent [7,16]. Ever since the discovery of the connection between NAD^+^ maintenance and the metabolic consequences of its decline in age-related and metabolic diseases such as obesity, diabetes, cardiovascular, Parkinson’s, and Alzheimer’s disease [17,18,19,20,21,22], approaches to increasing NAD^+^ levels have become therapeutic pursuits [23,24]. Some pre-clinical and clinical studies have sought to restore declining NAD^+^ levels by interference with NAD^+^-consuming processes (e.g., CD38 inhibitors [25,26] or PARP inhibitors [27]), by preventing the loss of NAD^+^ biosynthetic intermediates [28], by boosting nicotinamide conversion to NAD^+^ [29,30,31], or by supplementation with NAD^+^ precursors [32,33]. NAD^+^ precursors include nicotinamide [19], niacin [34], niacin derivatives [35,36], nicotinamide riboside (NR) [37,38,39], nicotinamide mononucleotide (NMN) [40,41,42,43], and the reduced forms of NR (NRH) and NMN (NMNH) [44,45,46,47]. From these precursors, NAD^+^ can be synthesized by three major biosynthetic pathways: (1) the de novo pathway or kynurenine pathway from tryptophan; (2) the Preiss–Handler pathway from niacin; and (3) the salvage pathway from nicotinamide. Two additional pathways to NAD^+^ uncovered more recently employ ribosylated biosynthetic intermediates. The first employs an NR kinase to generate NMN from NR [48,49,50], while the other pathway employs the reduced form of NR, NRH, and adenosine kinase for its phosphorylation to NMNH [13,44,51,52,53] (Figure 1). NMN and NMNH have also been shown to increase NAD^+^ levels in both cells and animal models although some controversy remains as to the feasibility of nucleotide transport across cellular membranes [54,55,56]. In addition to the presence of numerous phosphatases that convert nucleotides to nucleosides [57], the equilibrative nucleoside transporters (Figure 1) regulate the levels of nucleoside supplements that make their way to the intracellular space [58], while expression of kinases and hydrolysis of NR by purine nucleoside phosphorylase [59] and BST-1 [60] control the pathway by which NAD^+^ precursors truly affect NAD^+^ and NADH intracellular levels of a cell [61]. 

**Figure 2:**
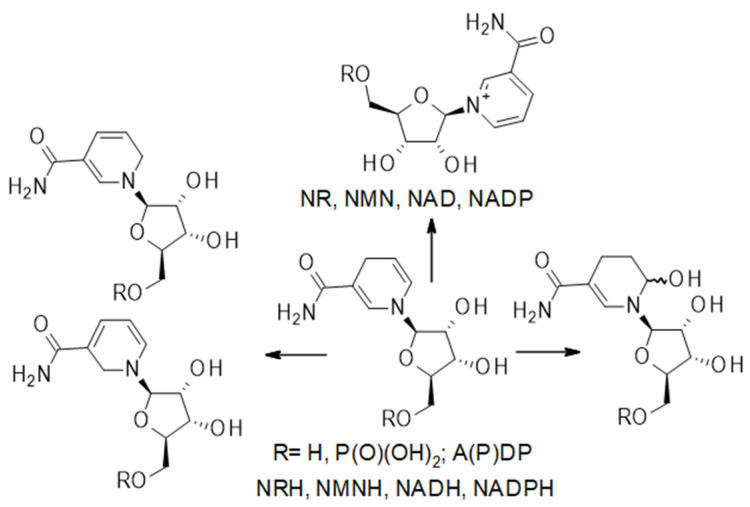
Schematic representation of NRH, NMNH, NADH and NADPH degradation pathways.

Challenges of measuring levels of precursors and NAD(P)(H) in biospecimens: It should be noted that NRH is readily oxidized in the presence of riboflavin, and free riboflavin cofactors and other oxidants are readily present in cells and tissue extracts, via processes that do not require enzymatic catalysis [62]. This oxidation process also occurs readily upon storage, even at low-temperature, and can also convert NMNH to NMN, NADH to NAD^+^, and NADPH to NADP^+^ (Figure 2). This oxidative process is often overlooked in the measurements of the NAD^+^ metabolome. Yet, on many occasions, only the NAD^+^ levels are reported, and it is unclear whether NAD^+^ from NADH oxidation contributes to this measurement. Similarly, acidic conditions applied during sample processing of biospecimens affect the levels of NADH and NADPH detected. Under acidic conditions, the reduced forms can degrade and thus does not contribute to the overall NAD(P)(H) measurements [63]. 

Considering the importance of total NAD(H) to cellular homeostasis (e.g., [64]), (NAD^+^ + NADH) levels should be measured simultaneously under conditions that conserve both NAD^+^ and NADH. Overall, conditions applied to sample processing and storage affect the measurements of the NAD(P)(H) metabolome and overall conclusions. As such, we recommend that the pH applied to samples is clearly conveyed and attention provided to reporting temperature and length of storage prior to sample processing. The addition of a labeled internal standard (e.g., isotopically labeled NAD(P)(H)), NR(H), or NMN(H)) added at the time of the sample collection would mitigate these experimental variables. 

Vitamin B3, like other B vitamins, must be ingested regularly to maintain functional levels of NAD(H) and NADP(H), together with NAD(P)(H), and is catabolized effectively via multiple pathways [65]. When used as supplements to redress declining levels in NAD(P)(H), supplementation with these precursors far exceeds (>100 mg/day) [47] the recommended daily dosage of vitamin B3 sought to be sufficient to maintain NAD^+^ levels and prevent pellagra (17 mg/day for an adult male) [66]. The premise for such high dosage administrations is that NAD^+^ boosting is transient, and that NAD^+^ precursors used even at high dosages are safe. Yet, each one of these NAD^+^ precursors is ultimately degraded, with the assumption that the final catabolites are excreted [67,68,69] without physiological interference. While some metabolites of NAD^+^ degradation (i.e., catabolites) are known [70], much of NAD(P)(H) catabolism remains uncharted. The catabolites that are known and measured see their abundance and distribution change depending on the precursor applied, the animal model, the clinical condition being investigated, and the biospecimens being measured [68,71,72,73]. Overall, the nature and the effects of catabolites of NAD^+^ and NADH and their sustained endogenous increase in disease or upon supplementation remain poorly understood, although concerns have been raised [73,74,75,76,77]. This warrants the need for more robust identification and characterization of the catabolites of NAD(P)(H). Here, we highlight these entities and provide a brief overview of the mechanisms which lead to their formation, and summarize biological observations directly related to their accumulation. 

## 3. Non-Ribosylated Catabolites of NAD(P)(H)

Primary catabolites of nicotinic acid and nicotinamide: Nicotinic acid and nicotinamide obtained from dietary sources are used for NAD^+^ synthesis, and most of the nicotinamide generated from NAD^+^ degradation is salvaged back to NAD^+^. However, excess nicotinamide, either released from orally administered NAD^+^ supplements (e.g., nicotinamide, NR, or NMN) or not recycled to NAD^+^, is subject to three major enzymatic clearance pathways. Nicotinamide is a substrate for microbial deamidases and can be converted to nicotinic acid by the microbiome [78]. In the mammalian gut, nicotinic acid generated from nicotinamide is adsorbed, enters circulation, and is promptly converted to NAD^+^ [78,79]. In the liver, excess nicotinic acid does not enter the NAD^+^ Preiss–Handler pathway and is metabolized to nicotinuric acid (NUA) by conjugation with glycine [80]. NUA (Table 1) is found in urine and is particularly abundant following nicotinic acid intake [81,82]. It has been proposed that changes in NUA levels can reveal an important pathogenic transition from metabolic syndrome to diabetes and atherosclerotic cardiovascular disease and thus be a potential marker of metabolic syndrome disease progression [83]. 

**Figure 3:**
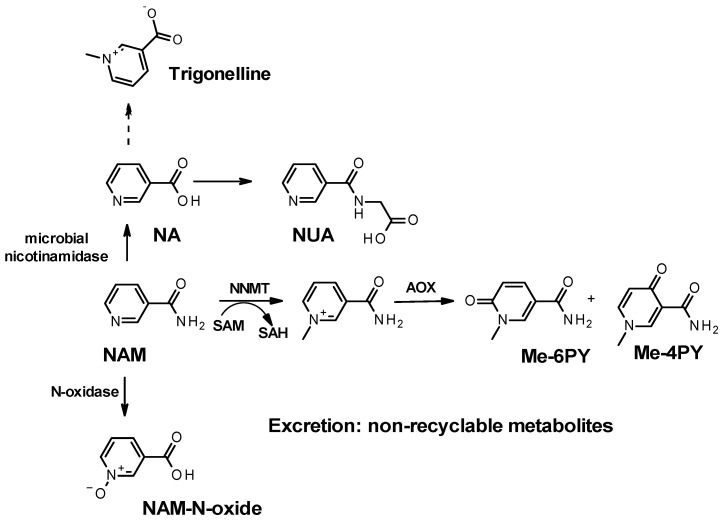
Known catabolites resulting from nicotinamide (NAM) catabolism. NA: nicotinic acid; NUA: nicotinuric acid; Me-6PY: *N*-methyl-3-carboxamide-6-pyridone; Me-4PY: *N*-methyl-3-carboxamide-4-pyridone; NAM-*N*-oxide: nicotinamide *N*-oxide; NNMT: nicotinamide *N*-methyltransferase.

If not converted to nicotinic acid, excess nicotinamide, either from dietary and supplement sources or from NAD^+^ degradation, can be oxidized to nicotinamide *N*-oxide (Figure 3) by CYP2E1 in the liver [84]. This end-product (Table 1) is found in circulation and as a urinary metabolite [85]. Interestingly, CYP2E1 is an enzyme that participates in the metabolism of other endogenous substrates, including acetone and fatty acids as well as exogenous compounds such as anesthetics, ethanol, nicotine, and acetaminophen. Excess nicotinamide might delay their metabolism. 

The most common degradation pathway for nicotinamide is methylation. Excess nicotinamide (from supplementation or increased NAD^+^ consumption) is often associated with increased plasma, serum, and urinary levels of *N*-Me-NAM (Table 1) [31,80]. Sun et al., demonstrated that excess nicotinamide led to catecholamine degradation in hypertensive mice resulting from the perturbation of the methylation pool and ultimately leading to enhanced levels of nicotinamide, homocysteine, and norepinephrine [77]. The enzyme responsible for the methylation of nicotinamide is now known as nicotinamide *N*-methyl transferase (NNMT) [86]. NNMT uses S-adenosylmethionine (SAM) as a co-substrate and methyl donor and generates *N*-methyl-nicotinamide (N-Me-NAM) and S-adenosyl homocysteine (SAH) (Figure 3), which is ultimately converted to homocysteine. Excess NAM and increased levels of *N*-Me-NAM have been associated with hyper-homocysteinemia and cardiovascular diseases, although the vasoprotective, anti-inflammatory, and anti-thrombotic roles of *N*-Me-NAM have also been documented [87]. More recent work indicates that methyl-nicotinamide might have a beneficial effect on cancer and cancer metastasis [88], while NNMT was shown to protect against oxidative stress-induced endothelial injury [89]. Alternatively, the progression of chronic kidney diseases is associated with a trend toward an increase in methylated catabolites of nicotinamide, whereby NNMT expression induces NAD^+^ and methionine metabolism perturbation contributing to renal and hepatic fibrosis [90,91]. NNMT hyper-activity not only affects NAD^+^ and SAM levels but can also redirect epigenomics and epi transcriptomics events [77,92]. However, a recent publication reported that NR supplementation (1 g/day) is not associated with altered methylation homeostasis in Parkinson’s disease [93]. Other studies have shown that nicotinamide supplementation resulted in a dose-dependent increase in oxidative stress and 8-hydroxy-2′-deoxyguanosine (8-OHdG)-positive cells both in the liver and kidneys and correlated with NNMT activity [74].

**Figure 4:**
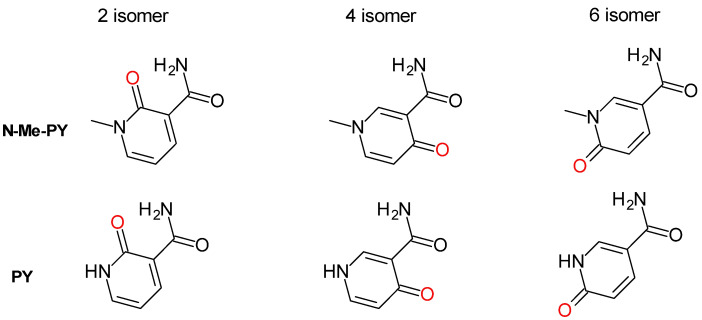
Pyridone catabolites derived from the nicotinamide scaffold.

Another methylated vitamin B3 derivative is trigonelline (Table 1). Trigonelline (Figure 4) is methylated nicotinic acid. Although not a known catabolite or precursor of NAD or NAAD, trigonelline is thought to have beneficial effects on human health. It is generated in plants by methylation of nicotinic acid, a process that mammalian NMNT is not known to carry out [94]. It is found in greater abundance in coffee beans, for which the levels increase upon torrefaction [95]. Trigonelline is not perceived to be a mammalian metabolite. However, it is often found in urinary specimens of coffee drinkers. A risk assessment of trigonelline consumption was recently conducted [96]. Although there was no evidence of adverse effects after acute exposure, no conclusion could be drawn on chronic exposure to isolated trigonelline due to the lack of data. Yet, trigonelline ingested as a component of coffee or coffee by-products was concluded to be safe for human health [96]. The effects of trigonelline on the overall levels of NAD(P)(H) and its precursors in biospecimens remain unexplored.

While these examples of correlations between excess nicotinamide and diseases can be potentially ascribed to a modulation in the intracellular abundance of SAM and NAD^+^, the full picture of induced dysfunction concerning excess nicotinamide is not yet acquired. Therefore, the need for monitoring the NAD^+^ catabolites of nicotinic acid and nicotinamide more systematically is warranted. For example, urinary and serum nicotinuric acid inform how well nicotinic acid is scavenged from microbial sources and used to generate systemic NAD^+^ via the Preiss–Handler pathway. Additionally, the accumulation of circulating and excreted methylated nicotinamide catabolites informs how much NAD^+^ consumption is required to maintain cellular homeostasis, which informs on the activity of sirtuins, ARTs, SARM1, BST1, and CD38. Similarly, the relative abundance of NAM-*N*-oxide, *N*-Me-NAM, and NUA compared with circulating NAM and NA is an indicator of the relative contribution made by the salvage and the Preiss–Handler pathways while maintaining intracellular NAD^+^ pools. Overall, a relative abundance of nicotinuric acid, nicotinamide N-oxide, and methyl-nicotinamide catabolites offer a window into systemic NAD^+^ metabolism and the use of its precursors for NAD(P)(H) level maintenance.

Oxidation of NAD(P)(H) primary catabolites: Among metabolic reactions, the oxidation of heteroaromatics accounts for the most predominant bioconversion in drug metabolism. Degradation of NAD^+^ metabolites through oxidation is therefore common and these species are the most encountered NAD^+^ catabolites in biospecimens [97]. The position of the resulting carbonyl with respect to the carboxamide of nicotinamide indicates the isomer formed upon oxidation. In mice, the 6 and the 4 isomers (as depicted in Figure 3) are the most frequently found isomers. In humans, the isomer Me-6-PY is most often reported as being the only detected isomer, and thus thought to be the predominant N-Me-NAM catabolite. Critically, Me-6-PY (Figure 3) is most frequently reported in the literature as 2-PY or Me-2-PY. This later nomenclature is particularly confusing since non-methylated oxidized nicotinamide, also labeled PY, can be detected in biological samples, although their levels are substantially lower than that of the methylated species. Furthermore, the nomenclature 2PY (aka. 6-Me-PY) positions the carboxamide of nicotinamide at the C5 of the pyridine ring. In nicotinamide, the carboxamide is located at the C3 of the pyridine ring. To avoid nomenclature confusion, we retain the same numbering for the carboxamide moiety on the oxidized pyridine ring as for nicotinamide with the pyridine nitrogen numbered as 1 and the carboxamide at position 3. As such, for all the isomers discussed henceforth, the carboxamide position can be mapped to that of nicotinamide and methyl-nicotinamide. 

It has been established that in mice, N-Me-NAM, like other arylamines, is oxidized by aldehyde oxidases [98] to generate these methyl pyridones (Figure 4; Table 1) and that this oxidative process can associate with the onset of Type 2 diabetes [99]. Pyridone derivatives of *N*-methyl-nicotinamide are major urinary metabolites of nicotinamide in humans and most mammals studied (comprehensively reviewed by Lenglet in 2018 [100]). Briefly, in humans, the *N*-methyl-2-pyridone-5-carboxamide, hence referred to as *N*-methyl-3-carboxamide-6-pyridone, (Figure 3 and Figure 4, N-Me-6-PY), is the predominant urinary end-product of NAD^+^ degradation to nicotinamide by NAD^+^-consuming enzymes, and nicotinamide surplus. In murine models, the *N*-methyl-3-carboxamide-4-pyridone (Figure 3 and Figure 4, *N*-Me-4-PY) is the most abundant catabolite [101]. Mammalian aldehyde oxidases (AOX) oxidize *N*-methyl-nicotinamide to the N-Me-4-PY and N-Me-6-PY [98]. These metabolites and the less abundant *N*-methyl-3-carboxamide-2-pyridone (Figure 4, N-Me-2-PY) have been proposed to be generated from the breakdown of NAD and NADP, rather than excess nicotinamide, although no evidence has been provided to support such a mechanism [101,102]. Other metabolites of nicotinamide include the less often measured 6-hydroxy-nicotinamide, also known as 6-hydroxy-3-carboxamide pyridine, 6-OH-Nam, or 6-PY (Table 1) (Figure 3), the non-methylated form of Me-6-PY [103]. However, the non-methylated pyridone 2-PY and the 4-PY (Table 1; Figure 4) may also be present but not measured. 

In mice administered nicotinamide or nicotinic acid, the 4 and 6 isomers of Me-PY (Figure 4) can be readily detected and are most abundant when the nicotinamide dose is in excess compared with that of nicotinic acid (NA), indicating that the mice microbiome can only handle so much nicotinamide before it releases it for use by the host [80]. In humans, Me-6-PY is often a major metabolite observed in urine and serum of subjects administered NAD^+^ precursors like nicotinamide [80], NR, and NMN [37,104]. Interestingly, Mierzejewska demonstrated that nicotinamide catabolites can serve as biomarkers to study the pathogenesis of bladder cancer [105]. They revealed that the concentration of *N*-Me-NAM was considerably decreased in bladder cancer patients with a concomitant increase in the NAM metabolites such as Me-6-PY. Nicotinamide end-products, as well as nicotinamide itself, are present in human and rat plasma, urine, whole blood, and erythrocytes, and their concentrations are elevated in animals with experimental chronic renal failure [68]. In mice, the plasma concentration of *N*-Me-4-PY is higher than that of *N*-Me-6-PY. This finding is contrary to that in humans with chronic renal failure where *N*-Me-6-PY is the predominant catabolite. Surprisingly, chronic kidney disease (CKD) patients show increased levels of *N*-methyl-4-pyridone-carboxamide (Me-4-PY) in addition to that of Me-6-PY [97,105,106,107,108], although Me-4-PY is not a known product of human AOXs [98,100]. Rutkowski et al. also demonstrated in rats that NAM end products can accumulate in different tissues and can ultimately lead to multiorgan impairment in the uremic state [71]. Importantly, Me-6-PY was shown to inhibit PARP in vitro and therefore potentially affect DNA repair capacity in CKD patients [109]. For further details on the subject, Lenglet and Massy provided a comprehensive review of the catabolites of methyl nicotinamide in 2016 [100]. 

The 2, 4, and 6 isomers of Me-PY can be synthesized [70] and used as standards in analyses by liquid chromatography coupled with mass spectrometry (LC-MS). Under such circumstances, it is more likely that all three isomers can be found and quantified in a biospecimen without pre-established assumptions. We found that in untargeted LC-MS, the Me-2-PY and Me-4-PY tend to co-elute unless the elution method is specifically optimized for their separation, but at the detriment of other catabolites. It is therefore easy to misconstrue their identity. Yet we must succeed in differentiating them if we are to characterize the pharmacological effects of endogenous, intracellular Me-PYs. For instance, AOX1 was shown both in animals and in vitro to catalyze the oxidation of Me-NAM and the formation of Me-4-PY [98]. On the other hand, the enzymatic conversion of Me-NAM to Me-2-PY and Me-6-PY remains speculative [100]. Consequently, the relative circulating abundance of Me-NAM to Me-PY in serum or urinary samples should offer an indication of the overall oxidative catabolic capacity of the organism. With emerging studies on NAD supplementations in murine models and human clinical trials, measurements of Me-NAM and Me-6-PY have become more widely reported [110]. By all accounts, supplementation with an NAD^+^ precursor, be it NAM, NR, NMN, NRH, or NMNH, leads to a substantial increase in Me-NAM and even more so in Me-4-PY and Me-6-PY. Yet, the pharmacological information that can be garnered from these measurements is limited to the translational assumption that NAD precursors are converted linearly to these catabolites, overlooking the importance of NUA, and nicotinamide-N-oxide, and that of the non-methylated PY species. Furthermore, it is likely that the role of each one of these catabolites while being generated endogenously, are different from their function once they enter circulation. Just like for NAM, NR, NRH, NMN, and NMNH, circulating catabolites of NAD^+^ might be subject to active transport in certain tissues or activate extracellular signaling sequences where they might be beneficial or nefarious. As such, a more comprehensive understanding of their pharmacokinetic properties is warranted. 

## 4. Ribosylated Catabolites of NAD(P)(H)

**Figure 5:**
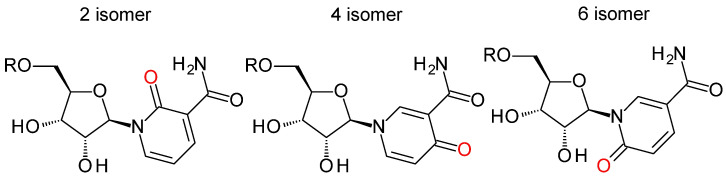
Hyperoxidized ribosylated catabolites of NAD(P). R = H, PYR; R = PO(OH)_2_, PYR-MP; R = PO(OH)OPO(OH)_2_, PYR-DP; R = PO(OH)OPO(OH)OPO(OH)_2_, PYR-TP; R = ADP; ox-NAD; R = APDP; ox-NADP.

While less often measured than Me-PYs, carboxamide pyridone ribosides (PYRs) have been detected in biological samples since the 1970s and were more formally characterized and isolated from human urine in the 1980s [111,112]. Once again, three isomeric forms exist based on the site of oxidation (Figure 5; Table 1). In human urine, the 1-ribosylpyridin-4-one-3-carboxamide (4-PYR; Figure 1) is found to be the most abundant circulating PYR [113]. The next most abundant isomer is 1-ribosylpyridin-6-one-3-carboxamide (6-PYR; Figure 5), also sometimes referred to 1-ribosylpyridin-2-one-5-carboxamide in the literature [114]. The last isomer is 1-ribosylpyridin-2-one-3-carboxamide (2-PYR), found to be much less abundant. Only a few of the PYR phosphorylated derivatives (Figure 5) have been described and characterized in the literature to date (Table 1). These derivatives include the nucleotide series, for which PYR can be mono, di, or triphosphorylated (PYR-NP), or conjugated to an adenosine diphosphate unit, as a pyridone adenine dinucleotide (ox-NAD; Figure 5) [111,114,115,116,117,118].

**Figure 6:**
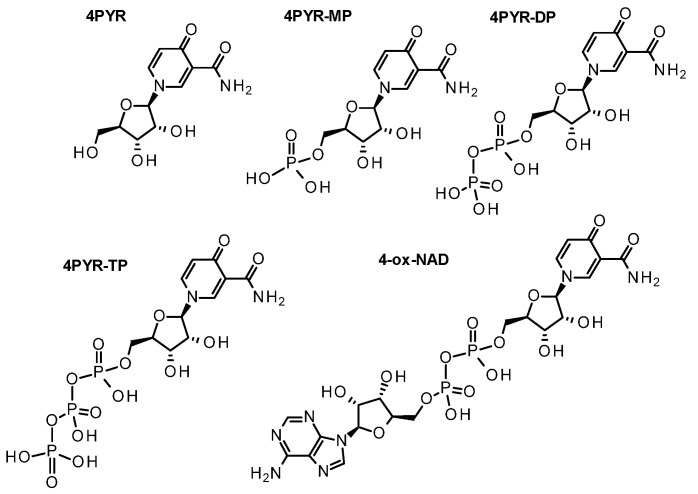
The 4-isomeric form of ribosylated pyridones, catabolites from nicotinamide riboside, nicotinamide mononucleotides, and NAD^+^.

Although PYR derivatives in bio-specimens have been quantified for more than 40 years, their origin remains mostly speculative. A biochemical relationship between the methylated (N-Me-PYs) and ribosylated (PYRs) forms of the pyridone species has been proposed but has yet to be identified. Instead, we posit that such a relationship does not exist and that PYRs were generated from the ribosylated forms of nicotinamide by over-oxidation of the pyridinium ring. The 2 and the 6 isomer ribosylated catabolites of NR, NMN, NAD, and NADP can be generated by Fenton chemistry [70]. However, this chemistry does not account for the formation of the 4 isomer. Alverti observed that an over-oxidized form of ox-NADP (Figure 5) could be generated by the flavin oxidoreductase, adrenodoxin (FDXR [119,120,121], while we observed that in the presence of oxygen, NQO2 could oxidize NRH but not NR to 4-PYR [62,122]. This observation indicates that electron transfer to oxygen via FADH_2_ is necessary and sufficient to enable the reaction between superoxide and pyridinium ring to generate the 4-hydroxylated pyridinium that then isomerizes to the pyridone. This mechanism is supported by the fact that NQO2 can generate superoxide [123], that can react with the pyridium ring of NR^+^. One can consider that NQO1, a component of complex I, might facilitate NAD^+^ over-oxidation (4-ox-NAD [124], Figure 6) under conditions that favor superoxide formation rather than electron transport during oxidative phosphorylation. The over-oxidation of NAD^+^ to 4-ox-NAD offers the platform for the formation of 4-PYR-MP via pyrophosphatase activity, and 4-PYR via phosphatases, offering a mechanism for the formation of circulating 4-PYR. Aside from the potential of 4-PYR as a possible indicator of tumor burden in malignancy [125], its abundance has been closely associated with the aging process and nephrotic dysfunction [126]. 4-PYR was also identified as one of the markers of good prognosis for survival in AIDS patients, and an independent predictor for AIDS progression [127]. Unlike the 2-PYR or the 6-PYR, this isomer of the pyridone series possesses a quinonoid structure that can act as an electrophile and interact with nucleophiles like cysteine and glutathione, in addition to DNA in a manner like a quinone. 

In the year 1979, Dutta and his team isolated and characterized 4-PYR from the urine samples of chronic myelogenic leukemia patients which was reported as the first pyridine-containing nucleoside derived from patient urine samples [112]. 4-PYR is commonly found in the plasma of healthy individuals in the nano-molar range (0.013 ± 0.006 μM). Increased concentrations of 4-PYR was found in the urinary excretions of several pathological conditions such as chronic renal failure [128], breast cancer [129], and chronic myelogenous leukemia patients [130] and was associated with tryptophan metabolism [116]. In patients with chronic renal failure, 4-PYR can accumulate substantially (>50 fold) [97]. Slominska and Rutkowski described the distribution of purine nucleotides in uremic fluids and tissues [131,132] and several studies have provided further insight into the abundance, role, and function of the PYR family.

Detailed investigations on this metabolite unveiled the fact that it becomes phosphorylated to its phosphate derivatives (Figure 6) such as 4-pyridone-3-carboxamide-1-β-D-ribonucleoside monophosphate (4-PYR-MP) and 4-pyridone-3-carboxamide-1-β-D-ribonucleoside triphosphate (4-PYR-TP) [115,117,131]. Multiple laboratories observed that in erythrocytes, extracellular 4-PYR was the precursor to intracellular 4-PYR-TP via 4-PYR-MP and adenosine kinase [117,118], although they observed a preferential accumulation of 4-PYR-MP over 4-PYR-TP during the incubation of 4-PYR. Later, Smolenski et al., observed that other tissues were able to metabolize, circulating 4-PYR to PYR-TP. They demonstrated that just like erythrocytes, other tissues could process 4-PYR including the liver, heart, kidneys, lungs, and skeletal muscles [113]. In addition, they showed that 4-PYR accumulated as 4-PYR-MP in all these tissues except the kidneys.

Measurements of 4-PYR-MP and 4-PYR-TP in human biospecimens revealed that 4-PYR-MP, and 4-PYR-TP are low in healthy adults but become elevated in the patients with chronic renal failure, which links the toxicity of these metabolites to renal anemia [132]. Furthermore, plasma concentration of 4-PYR in patients with chronic renal failure was found to be very high compared with that of healthy subjects. Initially, it was hypothesized that 4-PYR was either rapidly removed from circulation by renal clearance or converted to 4-PYR-TP at a faster rate in healthy subjects to clear this toxic metabolite from the plasma. The need for an effective renal clearance of 4-PYR would point to its possible toxicity and pathological conditions that might result from its accumulation. It was then proposed that prominent 4-PYR accumulation in circulation and peripheral tissues might further impair renal function.

To further investigate the effects of 4-PYR metabolites on the various metabolic processes that consume ATP or NAD, the effects of 4-PYR and its phosphorylated derivative 4-PYR-MP were evaluated against the enzymes involved in nucleotide metabolism and cellular metabolism [131,133,134]. While 4-PYR showed significant activation of S-adenosylhomocysteine hydrolase (SAHH), 4-PYR-MP was a potent inhibitor of adenosine monophosphate deaminase (AMPD) in erythrocyte lysate (IC_50_: 74 μM) and heart homogenates (IC_50_: 55 μM). Furthermore, the intracellular production of 4-PYR-MP from 4-PYR led to the inhibition of the AMP deamination pathway. It has been proposed that this inhibition contributes to the accumulation of adenine nucleotide observed in the erythrocytes of patients with chronic renal failure.

Using a widely studied animal model of atherosclerosis that shares many similarities to human pathology, Smolenski et al. observed that mice exposed to 4-PYR exhibited an increased deposition of lipids in their aortas as indicated by an increased area of atherosclerotic plaques in the abdominal region [126,135]. Furthermore, circulating 4-PYR accelerated atherosclerosis in these mice [136]. Extracellular adenosine deaminase activity was also enhanced upon 4-PYR treatment, decreasing intravascular adenosine levels. Wistar rats’ hearts perfused with 4-PYR were used to evaluate the 4-PYR metabolic pathways and discovered that 4-PYR was a precursor to yet another metabolite, 4-ox-NAD (Figure 6) [133]. 4-PYR-TP (Figure 6) and 4-ox-NAD could be detected in tissues following a 5 min exposure with 4-PYR solution, indicating a very effective uptake of circulating 4-PYR by tissues and conversion to nucleotides (Figure 1). Short-term exposure to 4-PYR on rat hearts did not affect the heart functions. 4-PYR had no acute cardiovascular toxicity but prolonged exposure to 4-PYR adversely affected the metabolism of endothelial cells, a process that has been proposed to lead to atherosclerosis. Slominska et al. later reported on the impact of 4-PYR metabolism on cellular energetic balance in endothelial cells which [134] included a decrease in NAD^+^ levels upon exposure to 4-PYR. Inhibition of the ENT transporter by dipyridamole abrogated these effects. Overall, 4-PYR conversion to its triphosphate and adenine dinucleotide was shown to have an adverse effect on energy balance in endothelial cells [137].

Crucially, 4-PYR-MP is converted to 4-ox-NAD via the NAD^+^ biosynthetic enzyme, NMNAT. This conversion was observed in human neuroblastoma cells, human malignant melanoma cells, stem cells derived from human adipose and bone marrow, human dermal microvascular endothelial cells, and human embryonic kidney cells [137]. Although 4-PYR was not shown to affect mitochondrial function, it was found to be detrimental to glycolysis and overall cellular bioenergetics [137]. In an NMNAT3 KD experiment, ATP, NAD, 4-PYR-MP, and 4-ox-NAD levels were affected by 4-PYR exposure. Surprisingly, 4-PYR-MP did not accumulate in these cells. This would be indicative of a very dynamic 4-PYR-MP metabolism, whereby 4-PYR-MP can either be hydrolyzed back to 4-PYR or metabolized to the triphosphate (PYR-TP). Unfortunately, these species were not measured in these experiments. Under NT5C2 KD conditions, the same cells responded to 4-PYR treatment with a 2-fold increase in 4-PYR-MP levels and a trend towards the increase in 4-ox-NAD levels. Importantly, ATP levels decreased to similar levels to that observed when NMNAT-3 KD was applied, linking increased ox-NAD levels to disturbance of bioenergetics.

Important developments associated with the physiological role of 4-PYR in the context of cancer have emerged more recently, whereby a higher concentration of 4-PYR is observed in the plasma of non-small cell lung cancer patients. Furthermore, an association between higher plasma 4-PYR concentrations and endothelial damage was observed in lung cancer patients. It was then proposed that the observed toxicity of 4-PYR towards the endothelium could lead to cancer cell proliferation, invasiveness, and inflammatory signaling [138]. Since then, circulating levels of 4-PYR and its metabolites have been closely associated with nicotinamide metabolism in bladder and breast cancer [105,139] and shown to correlate with breast cancer metastasis.

## 5. Discussion

There is growing evidence that NAD(P)(H) catabolites increase upon supplementation with NAD precursors, and that their distribution can inform on certain physiological endpoints and pathological progressions. In general, non-phosphorylated species are detected most reliably in extracellular matrices (e.g., serum, plasma, and urine; Table 1), while metabolites that are phosphorylated, e.g., nucleotides, are usually found intracellularly (e.g., whole blood and tissues; Table 1). When phosphorylated species are found in biospecimens that do not include cells or tissues, one should consider the possibility of lysis prior to or upon biospecimen sample collection rather than anticipate the presence of substantial levels of circulating nucleotides as contributing factors to the measurements.

Not only do circulating precursors and functional NAD(P)^+^ and NAD(P)H levels need to be reliably measured and benchmarked, identification and quantification of their respective catabolites should be included in such systematic reporting. Overall, the important role of NAD(P)(H) catabolism has mainly gone unnoticed and is often referred to as a correlation when diagnosing an underlying cause of a disease or monitoring the effect of supplementation. These catabolites can be biomarkers of disease and disease progression or potentially even healthy aging. It is now clear that the NAD(P)(H) metabolome is growing. Unfortunately, the identification, characterization, and quantification of new as well as known catabolites is limited by the chemical standards that are available to the analysts seeking their measurements. We generated Table 1 to provide a list of the known metabolites of nicotinamide and nicotinic acid that have been measured by LC-MS in biospecimens (except NAADP and NAADPH, as these remain elusive metabolites). For detection by mass spectrometry, most metabolites and catabolites respond well to positive ionization with a M+H^+^ ion. To help with this process, we provided the molecular formula of each molecular entity in Table 1. We hope that it advances the field in generating a more complete picture of the NAD(P)(H) metabolome with the view of exploiting it as a systemic biomarker of health.

## 6. Conclusions

There is no question that vitamin B3 is essential for human health. The observation that NAD+ declines with age coupled with the fact that NAD+ precursors (vitamin B3s included) need to be obtained through the diet have led to increased interest in supplementation. Furthermore, NAD^+^ boosting, enabled by a flurry of NAD^+^-boosting strategies in heathy and diseased states, has garnered increased attention worldwide. Yet, the balance that needs to be struck between increasing NAD^+^ levels and maintaining a healthy NAD(P)^+^ to NAD(P)H ratio relies on a complex network of gene regulation and protein expression that has yet to be fully unraveled. Although boosting NAD^+^ levels has gained scientific recognition over the past decades for its potential to address metabolism-related disorders, a possible concomitant increase in intracellular and circulating NAD^+^ catabolites can unexpectedly affect cellular and systemic homeostasis, and blur the functional gains achieved through NAD^+^ boosting. Although the methylated catabolites of nicotinamide are consistently measured in serum, blood, and tissues of interest, many other catabolites usually go unreported. Yet, they too have a story to tell. Overall, there is a dire need to investigate the physiological role of NAD^+^ catabolites as they are endogenously generated. The current state-of-the-art methods that assess the vitamin B3 metabolome to predict NAD^+^-derived biology remain limited in the context of NAD^+^ catabolites’ quantifications. The entire approach to measuring the NAD metabolome requires improvements. If their detection was standardized and cross-referenced with other markers of dysfunction, much can be unraveled from the status of the NAD(P)(H) metabolome and use of NAD^+^ precursors in healthy and diseased states. Understanding the type of catabolites generated and the ensuing biological consequence of their formation and circulation can open more astute treatment and functional supplementation programs.

## Figures and Tables

**Table 1:** List of known NAD(P)(H) precursors, biosynthetic intermediates, and catabolites, their molecular formula, and the biospecimens they are most likely detected from. Shaded colors indicate the possibility of NRH, NMNH, NADH, and NADPH contributing to the measurement of the NR, NMN, NAD^+^, and NADP^+^ pools.

		Name	Abbreviation in Text	Formula	Measurable in				Reported in			
					B	S/P	T	U	B	S/P	T	U
**Niacinamide/nicotinamide**	anabolites	nicotinamide	NAM	C_6_H_6_N_2_O								
		nicotinamide riboside	NR	C_11_H_15_N_2_O_5_^+^								
		nicotinamide riboside, reduced form	NRH	C_11_H_16_N_2_O_5_								
		nicotinamide mononucleotide	NMN	C_11_H_16_N_2_O_8_P^+^								
		nicotinamide mononucleotide, reduced form	NMNH	C_11_H_17_N_2_O_8_P								
		nicotinamide adenine dinucleotide	NAD	C_21_H_28_N_7_O_14_P_2_^+^								
		nicotinamide adenine dinucleotide, reduced form	NADH	C_21_H_29_N_7_O_14_P_2_								
		nicotinamide adenine dinucleotide phosphate	NADP	C_21_H_29_N_7_O_17_P_3_^+^								
		nicotinamide adenine dinucleotide phosphate, reduced form	NADPH	C_21_H_30_N_7_O_17_P_3_								
	catabolites	methyl-nicotinamide	*N*-Me-Nam	C_7_H_9_N_2_O^+^								
		methyl-2/4/6-pyridone	Me-2/4/6-PY	C_7_H_8_N_2_O_2_								
		2/4/6-hydroxy-nicotinamide	2/4/6-PY	C_6_H_6_N_2_O_2_								
		nicotinamide N-oxide	NAM-*N*-oxide	C_6_H_6_N_2_O_2_								
		2/4/6-pyridone carboxamide riboside	2/4/6-PYR	C_11_H_14_N_2_O_6_								
		2/4/6-pyridone carboxamide mononucleotide	2/4/6-PYR-MP	C_11_H_15_N_2_O_9_P								
		2/4/6-pyridone carboxamide riboside diphosphate	2/4/6-PYR-DP	C_11_H_16_N_2_O_12_P_2_								
		2/4/6-pyridone carboxamide riboside triphosphate	2/4/6-PYR-TP	C_11_H_17_N_2_O_15_P_3_								
		2/4/6-pyridone adenine dinucleotide	2/4/6-ox-NAD	C_21_H_27_N_7_O_15_P_2_								
		*2/4/6-pyridone adenine dinucleotide phosphate*	2/4/6-ox-NADP	C_21_H_28_N_7_O_18_P_3_								

		**Name**	**Abbreviation in text**	**Formula**	**Measurable in**				**Reported in**			
					**B**	**S/P**	**T**	**U**	**B**	**S/P**	**T**	**U**
**niacin**	anabolites	Nicotinic acid	NA	C_6_H_5_NO_2_								
		nicotinic acid riboside	NAR	C_11_H_14_NO_6_^+^								
		nicotinic acid mononucleotide	NAMN	C_11_H_15_NO_9_P^+^								
		nicotinic acid adenine dinucleotide	NAAD	C_21_H_27_N_6_O_15_P_2_^+^								
	catabolites	nicotinuric acid	NUA	C_8_H_8_N_2_O_3_								
		*trigonelline*	Trig	C_7_H_8_NO_2_^+^								

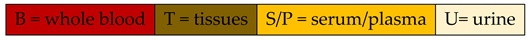
